# What Factors Have Been the Most Helpful and Harmful and When? Identifying Key Impacts on Psychosocial Development According to Autistic Adults and Caregivers

**DOI:** 10.1007/s10803-025-06800-4

**Published:** 2025-03-27

**Authors:** Juliette E. Lerner, Hillary Schiltz, Noa Schisterman, Sonja Ziegler, Catherine Lord

**Affiliations:** 1 Semel Institute for Neuroscience and Human Behavior, University of California, Los Angeles, 760 Westwood Plaza, Suite 68-217, Los Angeles, CA 90024 USA; 2 Department of Pediatrics in the Institute on Human Development and Disability, University of Washington, 1701 NE Columbia Road, Seattle, WA 98195 USA; 3 Research Unit of Child and Adolescent Mental Health, Department of Clinical Research, University of Southern Denmark, J.B Winsløws Vej 16, indgang 230, Odense, 5000 Denmark

**Keywords:** Autism, Lifespan, Family, Psychosocial development, Profound autism, Promotive and risk factors

## Abstract

Few studies have asked autistic adults and caregivers directly about what has most positively and negatively impacted their lives. This study sought to: (a) identify positive and negative factors experienced by autistic adults and caregivers; (b) test for within-subject differences in endorsement of promotive factors reported specific to four stages of development; and (c) test for differences in factors between adults with varying cognitive ability (i.e., less cognitively able [LCA; verbal IQ < 70] and more cognitively able [MCA; verbal IQ ≥ 70]). Participants included 91 autistic adults and caregivers. Autistic adults’ VIQs ranged from 4 to 139. Participants completed a modified version of the Social/Emotional Functioning Interview which consists of open-ended questions about positive and negative factors experienced across development. Autistic adults and caregivers, regardless of cognitive abilities, frequently reported people supports as more helpful than specific services, aspects of education, or generative activities from early childhood through adulthood. For both cognitive groups, generative activities were increasingly important after childhood. Services were more frequently identified as helpful by LCA caregivers in adulthood, while education was reported more by MCA caregivers and autistic adults. Differences by cognitive ability in negative factors included that more LCA caregivers reported poorly prepared professionals/caregivers as disruptive, while more MCA caregivers and autistic adults reported family conflict and bullying. Positive and negative factors identified through interviews of lived experiences can inform targeted care based on strengths and needs across cognitive abilities and life stages.

**Supplementary Information:**

The online version contains supplementary material available at 10.1007/s10803-025-06800-4.

Autism spectrum disorder (ASD) is a neurodevelopmental condition characterized by differences in social communication, interaction, learning, and behavior (American Psychiatric Association, 2013; Hodges et al., 2020). Autism is heterogeneous, not only in terms of presentation, but also in level of support needs and daily life experiences (Masi et al., 2017). While such heterogeneity is inherent in autism, positive and negative factors can shape the lives of autistic individuals and their families for better or for worse (Lord et al., 2020a; Lounds Taylor, 2017). Recent discourse highlights the importance of considering how constructs such as promotive and disruptive factors are defined, by whom, and for which ability and age group (Lam et al., 2021; McCauley et al., 2020; Pellicano & Heyworth, 2023). Historically, factors deemed helpful or harmful have been defined by researcher- and clinician-driven concepts of normative development (Pellicano & Houting, 2021). Through an often-used neurotypical lens, such factors also do not take into account autistic individuals’ or stakeholders’ appraisals of their success (Bishop-Fitzpatrick et al., 2016; Henninger & Taylor, 2013; Merrington et al., 2024; Ruble & Dalrymple, 1996). Consistent with the call to elevate autistic voices in autism research and practice (Lam et al., 2021; Pellicano et al., 2022), it is necessary to anchor this line of research within the lived experiences of autistic people and their families across a range of abilities and life stages (Georgiades & Kasari, 2018; McCauley et al., 2020; Robertson, 2009).

## The Case for First-Hand Accounts in Promotive and Disruptive Factors

Previous research has sought to characterize key experiences that either support or disrupt psychosocial trajectories in autism. Studies have identified that having formal and informal supports (i.e., services, professionals, interpersonal relationships and a close-knit community), engaging in vocational activities, and pursuing education can be particularly promotive for autistic individuals’ psychological well-being and later adjustment (Huang et al., 2022; Lord et al., 2020b; Schiltz et al., 2023). Conversely, negative factors such as systemic barriers (e.g., access to services), interpersonal issues (e.g., low social supports, bullying, victimization), and poor mental health have been found to be detrimental to quality of life and autonomy in adulthood (Libster et al., 2022; Lord et al., 2020b; Roestorf et al., 2022; Shattuck et al., 2020). A plurality of existing studies rely on observation and parent-report questionnaires (Frazier et al., 2023). Although these methods allow for replicability and comparisons of findings across studies (Henninger & Taylor, 2013), researchers and other members of the autism community have argued that many aspects of autistic success and experiences are missed when solely questionnaire-based and quantitative criteria are used (Gough & Madill, 2012; Mason et al., 2021; McConachie et al., 2018, 2020; Ruble & Dalrymple, 1996). A mixed-methods research approach, which leverages the strengths of both quantitative and qualitative analyses (Anguera et al., 2018; Creamer & Reeping, 2020), is essential for developing a more nuanced and comprehensive understanding of the factors affecting autistic individuals’ lives, particularly by incorporating perspectives rooted in lived experience.

## Accounting for Developmental Shifts and Cognitive Ability

Few autism studies consider multiple developmental stages when studying promotive and disruptive factors (Howlin & Moss, 2012; Mason et al., 2022; McCauley et al., 2020). Although there has been a recent increase in research studies that focus on ages beyond childhood, far less is known about autism into adulthood (Clark & Adams, 2021; Lord et al., 2020b; Mason et al., 2021; Roestorf et al., 2022). Change is experienced by autistic individuals and their families between and across developmental stages, including in autism features, adaptive skills, expectations and desires, and primary contexts like home, school, employment, social, and service settings (Clarke et al., 2021; Fountain et al., 2023; Lounds Taylor & Seltzer, 2010; Smith et al., 2012; Waizbard-Bartov & Miller, 2023; Woodman et al., 2016). As such, factors that contribute to challenges or flourishing at different developmental stages may change as well. For example, obstacles associated with the transition from adolescence to adulthood for autistic individuals, such as a poor person-environment fit and ambiguity surrounding the roles of caregivers (Anderson et al., 2018), may not be as relevant (or look the same) for a school-aged child transitioning into adolescence.

Further, individuals with lower cognitive and language abilities are generally underrepresented in the autism research literature (Russell et al., 2019; Stedman et al., 2018). Although current estimates place the co-occurrence of autism and intellectual disability (ID) at 30 to 40% (Maenner et al., 2023), only a small fraction of participants in autism research (i.e., 6% of the 100,245 autistic individuals across 301 studies published in 2016) have intelligence quotients (IQs) less than 70 (Russell et al., 2019). Individuals with profound autism–characterized by an IQ below 50, minimal or no verbal communication, and/or the need for 24-hour access to a caregiving adult (Lord et al., 2022)–are particularly under-represented in research (Thurm et al., 2021). This lack of inclusion is problematic, as there is not only considerable variability in the cognitive profiles of autistic individuals (Nowell et al., 2015), but also significant impacts of cognitive and language abilities on outcomes and trajectories among this population (Mason et al., 2021).

Among autistic individuals, IQ consistently predicts outcomes such as independent living, engagement in vocational and community activities, and having quality social contact (McCauley et al., 2020). Furthermore, a recent study that took place over three decades using an overlapping sample to the one in the present study found that those with profound autism had different outcomes in almost every domain–employment, independent living, friendships, and psychopathology (Lord et al., 2020b). However, little is known about how promotive and disruptive factors may differ across the spectrum of cognitive abilities.

## Current Study

To evaluate the factors which made the greatest positive and negative impacts on the lives of autistic individuals and their families across development, the current study sought to: (a) identify perceived positive and negative factors reported by autistic adults and their caregivers; (b) test for within-subject changes in endorsement of positive factors reported specific to four developmental stages; and (c) test for differences in positive and negative factors in caregiver reports for adults with differing cognitive ability (i.e., less cognitively able [LCA; verbal IQ < 70] and more cognitively able [MCA; verbal IQ ≥ 70]), as well as between MCA self- and caregiver-reports. The inclusion of caregiver reports in this study was particularly important for autistic adults such as those in the LCA group who were unable to provide interview responses. Additionally, incorporating caregiver reports for MCA autistic adults enabled comparisons between MCA autistic adults speaking for themselves and caregiver reports. We employed a mixed-methods study design consisting of two main steps: a content analysis of examiner-based interviews (qualitative approach) and tests of within- and between-subject differences (quantitative approach).

## Method

### Participants

The current study included 91 individuals with autism and related neurodevelopmental disorders (NDDs)^1^ enrolled in an ongoing longitudinal study. Respondents included 38 MCA autistic adults reporting on themselves, 40 parents reporting on MCA autistic adults, and 44 parents and one legal guardian (45 total) reporting on LCA autistic adults. Thirty-two of the MCA autistic adult participants have both a self and caregiver report; therefore, responses represent a total of 91 unique autistic adults across 123 reporters. Participants were originally recruited during childhood from community-based developmental clinics in North Carolina, the greater Chicago area, and Michigan. Participants in North Carolina and Chicago were recruited when participants were ages 2 to 3, and participants in Michigan were recruited when participants were approximately age 9. Data collection for the current study occurred when autistic adults were approximately 29 years old (*M* = 29.03, *SD* = 1.20) and caregivers averaged 60 years old (*M* = 60.07, *SD* = 6.30).

In the present sample, 18.7% of the autistic adults and 13% of caregivers were Black, and 17.6% of the autistic adults and 95.3% of caregivers were female. Compared to the full longitudinal cohort (*n* = 254, including attrited participants and those who were unable to participate in the current in-person visits), participants in this subset were significantly more likely to be White (*p* =.039) and to have maternal caregivers with at least a four-year college degree (*p* =.044). Participants in the full longitudinal cohort and the current subsample did not differ on any other demographic characteristics (i.e., participant sex, recruitment site, IQ, diagnosis of ASD or other NDD; all *p* >.05). Additional demographic information about this sample and the full longitudinal cohort is detailed in Table 1.

**Table 1:** Sample demographic characteristics

		SEF Autism Subsample	Total Autism Sample	X^2^ (df, *n*)	*p*
		*n* = 91 (%)	*n* = 254 (%)		
Sex	Male	75 (82.4)	204 (80.3)	0.19 (1, 345)	0.757
	Female	16 (17.6)	50 (19.7)		
Race	White/Caucasian	74 (81.3)	175 (69.2)	4.53 (1, 342)	0.039
	Black/African American	17 (18.7)	78 (30.8)		
Recruitment Site	North Carolina	43 (47.3)	132 (52.2)	0.90 (2, 344)	0.638
	Illinois	34 (37.4)	81 (32.0)		
	Michigan	14 (15.4)	40 (15.8)		
Caregiver Education	< Four-year degree	35 (39.3)	121 (53.3)	4.55 (1, 309)	0.044
	≥Four-year degree	54 (60.7)	106 (46.7)		
Diagnosis	Autism	76 (83.5)	196 (77.5)	1.48 (1, 344)	0.293
	Other NDD	15 (16.5)	57 (22.5)		
Cognitive Ability	Less Cognitively Able VIQ	45 (49.5) *M*_*VIQ*_ = 24	137 (58.5) *M*_*VIQ*_ = 26	2.20 (1, 325)	0.171
	More Cognitively Able VIQ	46 (50.5) *M*_*VIQ*_ = 104	97 (41.5) *M*_*VIQ*_ = 101		

*Note*. The current sample of autistic adults were approximately 29 years old (*M* = 29.03, *SD* = 1.20) and caregivers averaged 60 years old (*M* = 60.07, *SD* = 6.30) at the time of data collection. SEF = Social-Emotional Functioning Interview; NDD = neurodevelopmental disabilities; VIQ = verbal intelligence quotient from standardized measure. % = valid percent. IQs were collected at the most recent time point from when participants were seen as part of this study (*M* = 21.38 years, *SD* = 4.81)

**Verbal Cognitive Abilities**. Autistic adults’ verbal intelligence quotients (VIQs) ranged from 4 to 139, and therefore, consistent with previous research on this sample, participants in this study were grouped by cognitive abilities: LCA (VIQ < 70, *n* = 45, *M*_*VIQ*_ = 24, *SD* = 13.6) and MCA (VIQ ≥ 70, *n* = 46, *M*_*VIQ*_ = 104, *SD* = 18.9) (Anderson et al., 2014; McCauley et al., 2020). All participants in the LCA group had VIQs within the profound autism range (VIQ < 50) and 50% had VIQs less than 23. Approximately half of the MCA participants had VIQs in the average range (85–115), 30% were greater than 115, and about 20% had VIQs that fell between 68 and 84^2^. Verbal and nonverbal cognitive abilities were measured using developmentally appropriate, standardized cognitive assessments selected from the following: Wechsler Abbreviated Scale of Intelligence–Second Edition (WASI-II; Wechsler, 2011), Differential Abilities Scale–Second Edition (DAS-II; Elliott, 2007), or Mullen Scales of Early Learning (MSEL; Mullen, 1995). In cases where participants’ raw scores did not fall within standardized score ranges, ratio VIQs were calculated from age equivalents (see Anderson et al., 2014).

**Autism Diagnoses**. Diagnoses of ASD or other developmental disabilities were made by the research team based on observations (i.e., Autism Diagnostic Observation Schedule [ADOS-2]; Lord et al., 2012) and interviews (i.e., Autism Diagnostic Interview–Revised [ADI-R]; Lord et al., 1994) and confirmed by a panel of experienced clinicians with expertise in autism. Of the current participants, 15 had never received a formal ASD diagnosis despite early developmental delays. These participants were referred for autism evaluations in early childhood and remained in the analyses of this study because they have repeatedly demonstrated comparable profiles and outcome trajectories to those who received a formal diagnosis (Lord et al., 2020b; McCauley et al., 2020). Compared to participants with a formal autism diagnosis, those with other NDDs in this sample were significantly more likely to be female (*p* =.022), which is consistent with known sex-based differences in autism prevalence (Werling & Geschwind, 2013); however, they did not differ from those with a formal autism diagnosis across any other demographic characteristics (i.e., participant race, recruitment site, IQ, caregiver education, calibrated symptom severity scores from the ADOS-2; all *p* >.05).

### Procedure

Direct participant testing was completed in participants’ homes. Demographic and other questionnaires were completed either in-home, via mailed packets, or online. All participants were compensated following completion of in-person visits. Use of the current study’s data for research purposes was approved by and performed in compliance with the University of California, Los Angeles Institutional Review Board. Caregivers and, when possible, autistic adults provided written consent prior to each assessment.

### Measures

**Social/Emotional Functioning Interview–Subject or Informant Version (SEF-S & SEF-I**; adapted from Rutter et al., 1988). The SEF is a 90-minute examiner-based interview with structured and semi-structured items designed to gather information regarding a range of functioning areas, including housing and daily living activities, health and wellness, social and romantic relationships, and interests and future planning (Howlin et al., 2000; Rutter et al., 1988). Three versions of the SEF were used in the current study: a subject-report version completed by MCA autistic adults and informant-report versions specific to LCA and MCA adults that were completed by caregivers. Two SEF items asking participants to reflect on the most positive and negative factors experienced across the autistic adults’ development were the focus of the current study. See Table 2 for specific prompts and participant sample responses.

**Table 2:** Social-emotional functioning interview prompts and sample responses across self and caregiver reports

Positive Factors SEF Prompt	*What do you think made the most positive impact for them/you in their/your development? Was there a certain person(s) that helped significantly? Any particular activities, events, services, or things that helped you or your family? For this question, we’re going to break it up into different life stages…In early childhood, what made the biggest impact for them/you? In school age? In adolescence? Since becoming an adult?*
Category	Sample Responses Across Reporters
Services	**SR**: “Speech therapy,” “Getting diagnosed,” “Intervention,” “In-home therapy,” “Therapies,” “Circle time with peers,” “Vocational rehab,” “Easter Seals”
	**CR**: “Early childhood intervention,” “Home-based ABA,” “Desensitizing,” “Day program,” “Medical treatment for seizures,” “Group home,” “Medication”
People	**SR**: “Parents,” “Lifelong friends,” “Doctor,” “[Specifically named person],” “Siblings,” “Spending time with family,” “Grandma,” “Mentors,” “Having a girlfriend,” “Teacher in high school that encouraged me to go into acting”
	**CR**: “Family,” “Siblings,” “Mom and dad,” “Friend tribe,” “Grandparents,” “Providers were great,” “[Specifically named person],” “Teachers,” “Day caregiver,” “Full-time staff,” “Neighbors,” “Community workers,” “Job coach”
Education	**SR**: “Special education,” “IEP and accommodations,” “Education,” “Homeschool,” “Preschool for autism development,” “Associate degree”
	**CR**: “Mainstreamed,” “Special education classroom,” “Learning - school in general,” “Specialized school,” “College education,” “Master’s degree”
Generative Activities	**SR**: “Artistic hobbies,” “Jobs,” “Music,” “Gaming,” “Driving,” “Reading and writing,” “Public transportation,” “Moving out,” “Independence in college”
	**CR**: “Exercise,” “Getting a job,” “Developing own identity,” “Performing arts,” “Community integration,” “Having a routine,” “Drawing,” “Driver’s license”

Negative Factors SEF Prompt	*Are there any services, experiences, or people that had an especially negative impact? Is there anything that you think would have helped but never happened?*
Category	Sample Responses Across Reporters
Negative Impacts	**SR**: “Bullying, ostracized, general knowledge that I am ‘the other,’” “High standards of others at school,” “Mean supervisor at work,” “Disagreements with friends,” “Flunking college,” “Overweight”, “Never learned social skills,” “Family conflict,” “Confidence issues,” “Feeling isolated in college,” “OCD”
	**CR**: “Parents’ divorce,” “Aggression was major in early childhood,” “School system was unequipped, set him up to fail,” “Cut services/day program hours,” “Death of a family member,” “Anxiety,” “Big transitions,” “Bully at day camp,” “No special services,” “Self-injurious behavior,” “Abusive boss”
Things That Didn’t Happen but Would Have Helped	**SR**: “Put myself out there more,” “More free time doing stuff I enjoyed,” “Wish I had found something to be passionate about sooner,” “Graduating college,” “Friendships,” “Getting into therapy sooner,” “Relationship,” “More confident”
	**CR**: “No support system,” “Insisting on more speech therapy,” “More training for professionals,” “More services knowledge,” “Didn’t learn to read,” “Second set of support - sibling,” “Friendships and relationships,” “Driving,” “SSI”

*Note*. SEF = Social-Emotional Functioning Interview; SR = autistic adult self-report; CR = caregiver report

Positive factors responses were summarized and hand recorded by the interviewing clinician within the four major categories of *services*, *people*, *education*, and *generative activities* (defined further through qualitative content analyses described below) and delineated by developmental stage: early childhood, school age, adolescence, and adulthood. Negative factors responses were similarly hand recorded by the interviewing clinician during administration of the SEF within the two categories of *negative impacts* and *things that didn’t happen but would have helped (TTDH*). Notably, unlike the positive factors, inquiries about negative factors were not specific to a particular developmental stage unless the participant’s response spontaneously included such information (i.e., participant used temporal terminology such as “lack of services *in adulthood*” or “more *early* intervention”).

### Data Analytic Plan

#### Aim 1: Qualitative Coding of Open-Ended Responses

A team of coders used an iterative approach guided by qualitative content analysis (Elo & Kyngäs, 2008) to create a novel coding scheme of responses to the SEF items described above. This analytical process is multi-step and driven by the raw data of SEF responses which are delineated by developmental stage (for positive factors only) and by category. This entails a primarily inductive approach in which the coding team identifies codes directly from the data. As such, four coders reviewed the data independently and created a list of initial codes that captured the SEF responses (Step 1). These lists were then compared and revised collaboratively to form a consolidated codebook (Step 2). Then, the same four coders independently generated a grouping structure (i.e., subcategories) within *services*, *people*, *education*, and *generative activities* and within *negative impacts* and *TTDH (things that didn’t happen but would have helped)* based on commonalities among the codes (Step 3). Subsequently, the coding team met to discuss, come to a consensus, and define a comprehensive, integrated list of codes and subcategories (Step 4).

To maintain consistency and rigor in applying the coding scheme as described above, all positive factors data were double coded by alternating pairs of authors separately for each developmental stage. Negative factors data were coded by all four members of the coding team (rather than in pairs) due to their lack of separation by developmental stage and fewer data points. All four coding team members ultimately resolved discrepancies and agreed on the assignment of each code during the consensus meetings (i.e., no portion of the data were coded by a single person), with double-coded data ranging from 78 to 85% agreement even before consensus codes were assigned for each response.

To calculate frequencies of endorsements of positive and negative factors, participants (i.e., LCA caregivers, MCA caregivers, and MCA autistic adults) who provided a codable response received a 1 (endorsed) or 0 (did not endorse) for each code. Endorsement of positive factors at the category level refers to a binary code indicating whether at least one positive factor code (i.e., within *services*, *people*, *education*, and/or *generative activities*) was endorsed versus not endorsed. Primary analyses of positive factors were carried out at the combined category level; for comprehensiveness, details regarding lower-level subcategory and code frequencies are reported in Online Resource 1. Codes below 1% endorsement were excluded from the analyses.

#### Aims 2 & 3: Quantitative Tests for Differences by Developmental Stage and Reporting Group

**Positive Factors**. Data were analyzed using SPSS version 29.0. Multilevel logistic regressions were conducted to examine differences in the endorsement of positive factors across categories (i.e., *services*, *people*, *education*, *generative activities* within person), developmental stages (i.e., early childhood, school age, adolescence, adulthood within person), reporters (i.e., MCA autistic adults self-report vs. MCA caregiver report within person), and cognitive ability groups (i.e., between LCA vs. MCA caregivers). Multilevel models were selected over simple logistic regressions to account for correlations among repeated, non-independent observations through inclusion of a random intercept (e.g., the same individuals reported on four different developmental stages). We ran two models:

1. **LCA and MCA Caregivers**: This model included developmental stage (i.e., early childhood, school age, adolescence, adulthood), positive factors category (i.e., *services*, *people*, *education*, *generative activities*), cognitive ability (i.e., LCA vs. MCA), and their interactions. All variables are categorical and use dummy coding.
2. **MCA Autistic Adults and Caregivers**: This model included the same variables as above for developmental stage and positive factors category, but reporter status (i.e., MCA autistic adults self-report vs. caregiver-report) was included instead of cognitive ability group. See Online Resource 2 for a full list of model terms.

We also ran both models including demographic characteristics (i.e., participant sex, race, recruitment site, caregiver education level, diagnosis of ASD or other NDD, and calibrated symptom severity scores from the ADOS-2) to account for other potential explanatory variables. None of the demographic variables produced significant effects (all *p* >.05), nor did they alter the results of the overall models or target variables. Therefore, for simplicity’s sake, findings are reported for the models without demographic characteristics. Custom posthoc comparisons were conducted through estimated marginal means and pairwise contrasts using sequential Bonferroni corrections to account for multiple comparisons. The primary analyses of interest were interactions between developmental stage, positive factor categories, and cognitive ability group (i.e., LCA vs. MCA caregiver report) or reporter (i.e., MCA self- vs. caregiver-report).

**Negative Factors**. Whereas positive factors were delineated by developmental stage, negative factors were not queried in this way by the SEF item and therefore multilevel models were not required to account for non-independence of data. As such, frequencies of code endorsement were calculated, and chi-square tests were used to determine between-subject group differences in endorsement of codes and subcategories between cognitive ability groups (i.e., caregivers of LCA and MCA autistic adults). Separately, McNemar’s tests were used to test for differences across reporters (i.e., MCA autistic adult and MCA caregiver reports). All significance tests were two-tailed at *α* = 0.05.

### Positionality Statement

In conducting this study, it is important to recognize the influence of the authors’ backgrounds, experiences, and perspectives on qualitative coding processes. The first author (JL), who organized coding procedures, identifies as a middle-class White woman and sibling to an autistic adult. Among the other three coders, the first (HS) identifies as an upper-middle class White woman with two decades of experience and eight years of advanced training related to autism, the second (NS) as an upper-middle class White person and partner to an autistic adult, and the third (SZ) as a middle-class White woman with 17 years of experience and seven years of advanced training related to autism. None of the coders are autistic or caregivers of autistic individuals; however, their combined knowledge and expertise acquired through clinical training and practice, engagement with research participants and their families, and personal relationships with autistic individuals can serve to enrich the depth of the analyses and aid in ensuring that participants’ lived experiences are centered and valued. Although the coders made every effort to assign and reach consensus on codes in a rigorous manner, our individual backgrounds, cultural contexts, and prior experiences inevitably shape the lens with which we view the approach to data, identification of patterns, and interpretation of findings relevant to the current study.

## Results

Below we report frequencies of endorsement of common positive and negative factors using participants’ spontaneously generated and subsequently coded responses on the SEF (Aim 1). See Table 2 for example responses of autistic adult self-reports and caregiver reports, respectively. We report significant differences in the endorsement of positive factors at the category level (i.e., *services*, *people*, *education*, and *generative activities*) across developmental stages (i.e., early childhood, school age, adolescence, and adulthood), reporter (i.e., MCA autistic adults self-report vs. MCA caregiver report), and between cognitive ability groups (i.e., MCA vs. LCA caregivers) with a focus on interpreting interactions using posthoc pairwise comparisons from the multilevel logistic regressions (Aims 2 & 3). Finally, we report differences in negative factors between cognitive ability groups (i.e., MCA vs. LCA caregivers) and across reporters (i.e., MCA autistic adults self-report vs. MCA caregiver report) using chi-square and McNemar’s tests, respectively (Aim 3).

### Positive Factors

The first multilevel logistic regression model with LCA and MCA caregivers yielded a significant main effect of the positive factors category, *F*(3, 1176) = 32.64, *p* <.001, on frequency of endorsement (see Online Resource 2 and 3 for model coefficients and significance tests). Pairwise contrasts revealed that caregivers’ endorsement of *people* was significantly higher on average than all other categories of positive factors: *services*, *t*(1176) = 7.15, *p* <.001; *education*, *t*(1176) = 10.10, *p* <.001; and *generative activities*, *t*(1176) = 8.42, *p* <.001. Caregivers’ endorsement of *services* was significantly higher than *education*, *t*(1176) = 2.52, *p* =.035. There were no other significant main effects in this model. These effects were further characterized by significant interactions, including a two-way interaction between developmental stage and positive factors category, *F*(9, 1176) = 5.33, *p* <.001, and a three-way interaction between positive factors category, developmental stage, and cognitive ability group, *F*(15, 1176) = 2.27, *p* =.004. As such, to further probe this interaction, each positive factors category was examined separately; findings within each category are described in the sections below.

The second multilevel logistic regression model with MCA autistic adults and caregivers yielded a similar main effect and two-way interaction as the first model (i.e., significant main effect of positive factors category such that *people* was significantly higher than all other positive factors categories and significant two-way interaction between developmental stage and positive factors category, all *p* <.001). In this model, MCA autistic adult and caregivers’ endorsement of *generative activities* was significantly higher on average than *services*, *t*(1015) = 3.80, *p* <.001. The main effect of reporter and any interactions with reporter (e.g., interaction between positive factors category, developmental stage, and reporter) were non-significant. That is, MCA autistic adults’ and MCA caregivers’ endorsement of positive factors categories did not significantly differ from each other overall, and this pattern appeared consistent across developmental stages and for all positive factors categories.

**Fig. 1:**
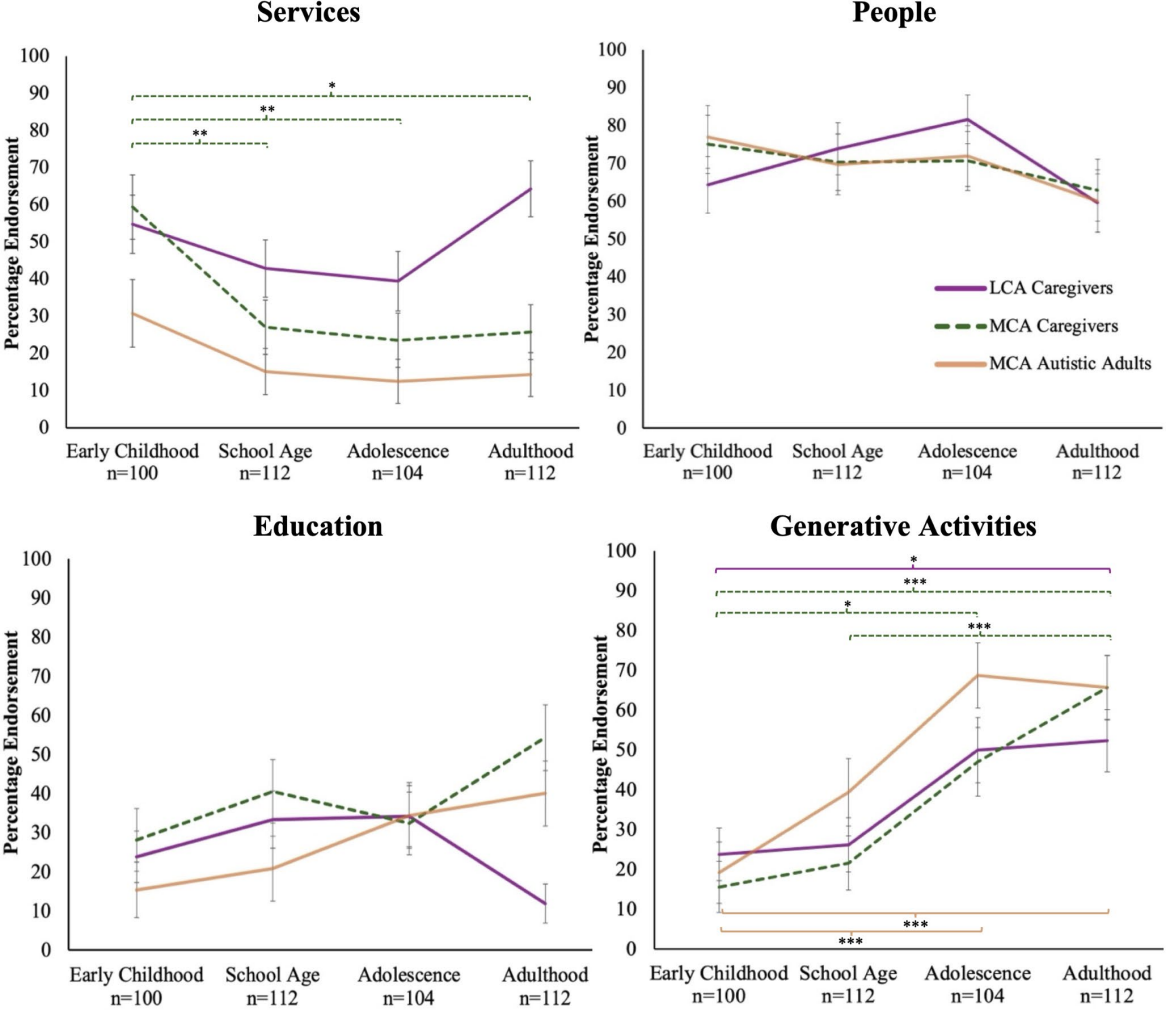
Endorsement of positive factors categories and differences across developmental stages. *Note*. Percentage endorsement refers to the proportion (converted to percent) of respondents who endorsed at least one code within the *services*, *people*, *education*, and *generative activities* categories reporting on each developmental stage. *Services* = intervention, assessment, community/educational programming; *people* = family, formal/informal people supports; *education* = school characteristics, schooling/learning in general; *generative activities* = vocational/leisure activities, related internal/external factors. Error bars represent standard error. MCA = more cognitively able; LCA = less cognitively able. *p <.05, **p <.01, ***p <.001

**Services**. Qualitative content analysis of the *services* category identified the following subcategories: intervention, assessment, programming, medical resources, and other structured service provisions (e.g., skills courses, accommodations/academic support). Overall, the percentage of caregivers who endorsed the *services* category ranged from 31 to 57% (*M* = 41.30, *SE* = 3.08) from early childhood to adulthood. See Fig. 1; Table 3 for endorsement of positive factors categories across developmental stages and between reporters, respectively.

**Table 3:** Endorsement of positive factors categories and differences by cognitive ability

	LCA Caregiver	MCA Caregiver	MCA Autistic Adult	LCA vs. MCA Caregiver Difference	
	*M% (SE)*	*M% (SE)*	*M% (SE)*	*t*	*p*
*Services*					
Early Childhood	54.76 (7.74)	59.38 (8.75)	30.61 (9.07)	− 0.40	0.690
School Age	42.86 (7.69)	27.03 (8.75)	15.09 (6.24)	1.48	0.139
Adolescence	39.47 (7.98)	23.53 (7.30)	12.48 (5.85)	1.46	0.144
Adulthood	64.29 (7.44)	25.71 (7.41)	14.25 (5.92)	3.69	**< 0.001**
*People*					
Early Childhood	64.29 (7.49)	75.00 (7.66)	76.83 (8.32)	−1.01	0.315
School Age	73.81 (6.86)	70.27 (7.53)	69.64 (8.05)	0.34	0.733
Adolescence	81.59 (6.37)	70.59 (7.83)	71.89 (7.98)	1.08	0.279
Adulthood	59.52 (7.67)	62.86 (8.18)	60.00 (8.33)	− 0.29	0.774
*Education*					
Early Childhood	23.81 (6.61)	28.13 (7.96)	15.28 (7.06)	− 0.42	0.674
School Age	33.33 (7.35)	40.54 (8.09)	20.61 (8.26)	− 0.67	0.504
Adolescence	34.21 (7.75)	32.35 (8.03)	34.36 (8.44)	0.154	0.877
Adulthood	11.90 (5.01)	54.29 (8.44)	39.96 (8.32)	−4.30	**< 0.001**
*Generative Activities*					
Early Childhood	23.81 (6.60)	15.63 (6.44)	19.12 (7.72)	0.88	0.377
School Age	26.19 (6.81)	21.62 (6.80)	39.30 (8.55)	0.47	0.641
Adolescence	50.00 (8.18)	47.06 (8.63)	68.76 (8.23)	0.24	0.813
Adulthood	52.38 (7.77)	65.71 (8.09)	65.70 (8.07)	−1.18	0.238

*Note*. LCA = less cognitively able; MCA = more cognitively able

To probe the three-way interaction noted above, pairwise comparisons were conducted across developmental stages for both cognitive ability groups. Findings showed that LCA caregivers’ endorsement of *services* did not differ significantly (all *p* >.05) across early childhood, school age, adolescence, and adulthood. For MCA caregivers, endorsement of *services* was significantly higher in early childhood compared to all other developmental stages: school age, *t*(1176) = 3.73, *p* =.001, adolescence, *t*(1176) = 3.19, *p* =.007, and adulthood, *t*(1176) = 2.97, *p* =.012 (Fig. 1). For sake of comprehensiveness, the interaction was also probed using pairwise comparisons between cognitive ability groups within each developmental stage. LCA caregivers significantly more often endorsed *services* in adulthood compared to MCA caregivers, *t*(1176) = 3.69, *p* <.001, but not at any other developmental stages (Table 3).

The percentage of MCA autistic adults who endorsed the *services* category ranged from 13 to 31% (*M* = 17.19, *SE* = 3.48) from early childhood to adulthood. Pairwise comparisons revealed no significant differences in MCA autistic adults’ endorsement of *services* across developmental stages (Fig. 1; Table 3).

**People**. Qualitative content analysis of the *people* category identified the subcategories: family, formal supports (e.g., professionals, teachers, other caregivers), and informal supports (e.g., friends/peers, romantic partners, pets, and other specifically named individuals). Overall, the percentage of caregivers who endorsed the *people* category ranged from 61 to 77% (*M* = 70.20, *SE* = 3.32) from early childhood to adulthood (Fig. 1; Table 3). Pairwise comparisons were examined and showed no significant differences in the endorsement of *people* across developmental stages for LCA or MCA caregivers. Likewise, there were no differences in the endorsement of *people* between LCA and MCA caregivers at any developmental stage.

The percentage of MCA autistic adults who endorsed the *people* category ranged from 60 to 77% (*M* = 69.91, *SE* = 4.25) from early childhood to adulthood. Pairwise comparisons revealed no significant differences in MCA autistic adults’ endorsement of *people* across developmental stages (Fig. 1; Table 3).

**Education**. Qualitative content analysis of the *education* category identified the subcategories: school characteristics (e.g., special education, general education), school type (e.g., homeschool, private school), and schooling/learning in general. Overall, the percentage of caregivers who endorsed the *education* category ranged from 26 to 37% (*M* = 30.93, *SE* = 2.86) from early childhood to adulthood (Fig. 1; Table 3). Pairwise contrasts across developmental stages revealed no significant differences in the endorsement of *education* for LCA or MCA caregivers. Examination of comparisons across cognitive ability groups revealed that MCA caregivers had a significantly higher endorsement of *education* in adulthood compared to LCA caregivers, *t*(1176) = 4.30, *p* <.001, but not at any other developmental stage (Table 3).

The percentage of MCA autistic adults who endorsed the *education* category ranged from 15 to 40% (*M* = 26.33, *SE* = 4.41) from early childhood to adulthood. Pairwise comparisons revealed no significant differences in MCA autistic adults’ endorsement of *education* across developmental stages (Fig. 1; Table 3).

**Generative Activities**. Qualitative content analysis of the *generative activities* category identified the subcategories: vocational or leisure activities (e.g., employment, hobbies/extracurriculars), individual/internal factors (e.g., outlook/attitudes, autonomy/independence), and environmental/external factors (e.g., structure/routine, transportation, finances). Overall, the percentage of caregivers who endorsed the *generative activities* category ranged from 19 to 59% (*M* = 36.09, *SE* = 3.03) from early childhood to adulthood (Fig. 1; Table 3).

Pairwise contrasts across developmental stages revealed that LCA caregivers’ endorsement of *generative activities* in adulthood was significantly higher than early childhood, *t*(1176) = 2.83, *p* =.028, and marginally higher than school age, *t*(1176) = 2.57, *p* =.052. Additionally, for LCA caregivers, endorsement of *generative activities* was marginally higher in adolescence compared to early childhood, *t*(1176) = 2.49, *p* =.052 (Fig. 1). For MCA caregivers, endorsement of *generative activities* in adulthood was significantly higher compared to early childhood, *t*(1176) = 4.87, *p* <.001, and school age, *t*(1176) = 4.19, *p* <.001. Additionally, for MCA caregivers, endorsement of *generative activities* in adolescence was significantly higher than early childhood, *t*(1176) = 2.93, *p* =.014, and marginally higher than school age, *t*(1176) = 2.32, *p* =.061 (Fig. 1). Contrasts across cognitive ability group showed no differences in the endorsement of *generative activities* between LCA and MCA caregivers at any developmental stage (Table 3).

The percentage of MCA autistic adults who endorsed *generative activities* ranged from 19 to 69% (*M* = 47.26, *SE* = 5.09) from early childhood to adulthood. Pairwise contrasts across developmental stages revealed that MCA autistic adults’ endorsement of *generative activities* was significantly higher in adolescence, *t*(1015) = 4.41, *p* <.001, and adulthood, *t*(1015) = 4.18, *p* <.001, compared to early childhood and marginally higher in adolescence compared to school age, *t*(1015) = 2.49, *p* =.052 (Fig. 1; Table 3).

### Negative Factors

**Negative Impacts**. Qualitative content analysis of the *negative impacts* category identified the subcategories: external social challenges, individual/internal challenges, medical/psychological challenges, caregiver challenges, systemic challenges, adverse events, and no perceived negative impacts. See Table 4 for a full list of negative factors subcategories, codes, frequencies, and significant group differences.

**Table 4:** Percentage of endorsed negative factors reflecting across the lifespan by reporter

Categories, Subcategories, & Codes	Negative Factors			
	All *n* = 109	LCA Caregiver *n* = 42	MCA Caregiver *n* = 36	MCA Autistic Adult *n* = 31
	%	%	%	%
**Category: Negative Impacts**				
*Subcategory: External Social Challenges*	33.94	28.57	38.89	35.48
Bullying/Victimization/Abuse/Getting Taken Advantage Of	12.84	7.14	^**†**^ **25.00**	^**†**^ **6.45**
Lack of Social Support/Isolation/Negative Social Interaction	13.76	14.29	5.56	22.58
Moving/Transitions (social or physiological)	9.17	9.52	8.33	9.68
*Subcategory: Individual/Internal Challenges*	30.28	23.81	38.90	29.03
School/Academic Difficulties	21.10	16.67	30.56	16.13
Social/Communication Challenges	3.67	2.38	0.00	9.68
Underdeveloped Adaptive/Social Skills	3.67	4.76	5.56	0.00
Lack of Confidence	2.75	0.00	2.78	6.45
*Subcategory: Medical/Behavioral Health Challenges*	19.27	21.43	11.11	25.81
Mental/Behavioral Health Condition or Problem/Self-Injurious Behavior	10.09	9.52	2.78	19.35
Medical Condition/Accident	8.26	11.90	8.33	3.23
Weight	1.83	2.38	2.78	3.23
*Subcategory: Caregiver Challenges*	17.43	7.14	**30.56**	16.13
Family Conflict/Issue with Parent(s)/Overbearing Caregiver	15.60	4.76	**27.78**	16.13
Caregiver Declining Health	2.75	4.76	2.78	0.00
*Subcategory: Systemic Challenges*	22.94	**38.10**	13.90	12.91
Poorly Prepared Professionals/Caregivers/Bad Program Experience	11.93	**23.81**	5.56	3.23
Medical Mismanagement/Lack of Medical Success	1.83	4.76	0.00	0.00
Issues at Place of Work or Living	7.34	9.52	2.78	9.68
Lack of Services	5.50	9.52	5.56	0.00
*Subcategory: Adverse Events*	21.10	11.90	25.00	29.03
Relationships Ending/Divorce	11.93	4.76	16.67	16.13
Death/Passing	8.26	4.76	11.11	9.68
Involvement with Law Enforcement	1.83	0.00	2.78	3.23
Getting Kicked Out of a Group or Program	2.75	4.76	2.78	0.00
*Subcategory: No Perceived Negative Impacts*	4.59	4.76	8.33	0.00
Nothing Negative/Harmful	4.59	4.76	8.33	0.00
**Category: Things That Didn’t Happen but Would Have Helped**				
*Subcategory: Individual Characteristics*	8.26	9.52	2.78	12.90
Better Social/Communication/Life Skills	2.75	4.76	0.00	3.23
Improved Self Confidence/Advocacy	1.83	0.00	0.00	6.45
Better Managing Health/Coping Strategies	3.67	4.76	2.78	3.23
*Subcategory: Better/More Services*	18.35	23.81	25.00	^**†**^ **3.23**
Better Equipped Teachers and Service Providers	9.17	11.90	11.11	3.23
More Guidance/Resources on Services	4.59	9.52	2.78	0.00
Psychoeducation on Autism/Development	1.83	0.00	5.56	0.00
Early Intervention	2.75	4.76	2.78	0.00
More Services in Adulthood	1.83	2.38	2.78	0.00
*Subcategory: More Generative Activities/Opportunities*	25.69	21.42	22.22	35.49
More Availability/Participation/Buy-In/Engagement in Services	9.17	9.52	13.89	3.23
Hobbies/Travel Sooner and More Developed	4.59	2.38	0.00	12.90
Personal Accomplishments and Sooner (educational, vocational, other)	9.17	9.52	8.33	9.68
More Fulfilling Job/Quality Employment and Sooner	2.75	0.00	0.00	9.68
*Subcategory: External Supports*	16.51	11.90	19.45	19.36
More Social Contact/Social Events/Romantic Relationships	8.26	2.38	13.89	9.68
More Social/Familial Support	2.75	7.14	0.00	0.00
Financial Support	5.50	2.38	5.56	9.68
*Subcategory: No Perceived Things That Didn’t Happen*	10.09	7.14	13.89	9.68
Nothing Helpful That Didn’t Happen	10.09	7.14	13.89	9.68

*Note*. **Bold** indicates significantly greater endorsement of code or subcategory between LCA and MCA caregiver reports at *p* <.05, ^**†**^**bold** indicates at *p* =.056 for the difference between LCA and MCA caregiver report and at *p* =.070 for the difference between MCA caregiver and MCA autistic adult report; missing or un-codable data varied from 6-16%; sample sizes and percentages reported above are excluding missing and un-codable data. MCA = more cognitively able; LCA = less cognitively able

The *external social challenges* (i.e., bullying/victimization, lack of social support, and moving/transitions) and *individual/internal challenges* (i.e., school/academic difficulties, issues with communication/adaptive skills, lack of confidence) subcategories were relatively consistently endorsed by 24 to 39% of participants across reporters and cognitive ability groups (MCA and LCA caregivers and MCA autistic adults). The most common *individual/internal challenges* code was *school/academic difficulties*, which was relatively consistent across cognitive ability groups and reporters (LCA caregiver: 17%, MCA caregiver: 31%, MCA autistic adult: 16%).

In contrast, specific *external social challenges* were described differently across cognitive ability groups and reporters. *Bullying/victimization* was endorsed by a marginally higher percentage of MCA caregivers compared to LCA caregivers (LCA = 7%, MCA = 25%, *p* =.056) and MCA caregivers compared to MCA autistic adults (MCA autistic adult = 6%, MCA caregiver = 25%, *p* =.070, Table 4). *Lack of social support/isolation* was an *individual/internal challenge* commonly endorsed by both LCA caregivers (14%) and MCA autistic adults (23%), but not as many MCA caregivers (6%), although this difference was not statistically significant.

The *medical/behavioral health challenges* subcategory (e.g., mental or behavioral health problem, challenging behavior, and medical condition/concern) was endorsed by approximately a quarter of LCA caregivers (21%) and MCA autistic adults (26%). Although not significant, MCA caregivers endorsed this subcategory less often than the other two groups (11%; Table 4).

The *caregiver challenges* subcategory (i.e., family conflict, caregiver declining health) was identified significantly more often as problematic by MCA caregivers (31%) than LCA caregivers (7%, *p* =.009) or MCA autistic adults (16%, although the difference in self- vs. caregiver-report was not significant). This difference appeared to be driven by differences in the *family conflict* code which was identified as a negative factor by significantly more MCA caregivers than LCA caregivers (LCA caregiver: 5%, MCA caregiver: 28%, *p* =.009), and, although not significant, more MCA caregivers than autistic adults (16%; Table 4).

The *systemic challenges* subcategory consisted of multiple codes including poorly prepared professionals/caregivers and bad experiences in specific programs, challenges with service settings, and workplace or housing issues. A significantly higher percentage of LCA caregivers (38%) endorsed the *systemic challenges* subcategory than MCA caregivers (14%; *p* =.021); this difference appeared to be driven by the *poorly prepared professionals/caregivers/bad program experience* code (LCA = 24%, MCA = 6%, *p* =.031; Table 4).

The *adverse events* subcategory (e.g., relationships ending or divorce, experiences with death, getting removed from a group or program) was reported as negative by approximately a fourth of MCA autistic adults (29%) and MCA caregivers (25%) and not as many LCA caregivers (12%), although these differences did not reach significance (Table 4).

There were small numbers of participants in both caregiver groups who endorsed the *no perceived negative impacts* code (5–8%). No MCA autistic adults reported this code (Table 4).

**Things That Didn’t Happen but Would Have Helped (TTDH)**. Qualitative content analysis of the *TTDH* category identified the subcategories: individual characteristics, better/more services, generative activities/opportunities, external supports, and no perceived things that didn’t happen. Overall, both the *better/more services* and *more generative activities/opportunities* subcategories were relatively consistently identified by 21 to 25% of MCA and LCA caregivers. MCA autistic adults had a marginally lower endorsement of *better/more services* compared to MCA caregivers (MCA autistic adult = 3%, MCA caregiver = 25%, *p* =.070). Of note, 7 to 14% of participants across reporting groups endorsed *no perceived things that didn’t happen* (LCA caregiver: 7%, MCA caregiver: 14%, MCA autistic adult: 10%; Table 4). There were no significant differences in *TTDH* subcategories and codes by cognitive ability groups (MCA vs. LCA caregivers).

## Discussion

These findings provide a novel characterization of promotive and disruptive factors experienced across developmental periods, as reflected on by autistic and NDD adults and their caregivers. Critically, these findings are aligned with the lived experiences of a cognitively diverse sample of autistic individuals, contributing to a growing literature base which includes a spectrum of capabilities in defining sources of strengths and support needs among this population. Further, the results underscore the importance of including perspectives from autistic individuals and their families to better understand the unique, dynamic, and multifaceted aspects of what may shape autistic individuals’ psychosocial development. Analyzing participants’ spontaneous responses to inquiries provided evidence of some differences between the perspectives of MCA and LCA caregivers, particularly as their children reached adulthood, with endorsement patterns unique to the positive factor category. For negative factors experienced across the lifespan, commonalities and differences by cognitive ability also emerged. There were relatively few differences between MCA autistic adults and MCA caregivers (all statistically small or non-significant) in their endorsement of positive and negative factors in this study, demonstrating their comparability as reporters of these aspects of autistic individuals’ lives.

### Positive Factors– Comparisons between Cognitive Ability Groups and across Development

Participants’ experiences of service systems (i.e., intervention, community and specific programming) and education (i.e., school settings and schooling and learning in general) exhibited differences by cognitive ability, particularly in adulthood. While services were highly endorsed across all stages for LCA caregivers, MCA adults and caregivers reported services to be most helpful in early childhood, with perceived helpfulness declining later in life. A nearly opposite pattern emerged for education such that endorsement was relatively stable until adulthood, at which point MCA caregivers and autistic adults reported education as helpful more often than LCA caregivers. Thus, while continuing or bolstering structured support systems during the transition to adulthood may be a priority for LCA autistic adults, not surprisingly, MCA autistic adults place particular value on post-secondary education.

Individuals with profound autism, represented within the LCA group in this study, have considerable service needs (Clarke et al., 2024; Ferguson et al., 2024), which include access to regular opportunities for meaningful socialization, primary health care with staff who are trained in autism, social and life skills interventions, and behavioral support (Ferguson et al., 2024). LCA caregivers may not only endorse services as more helpful but also receive more services in response to higher support needs. In an informal posthoc exploration using available background history questionnaires, 72% of caregivers in this sample reported that their child had accessed at least one service in adulthood (e.g., in-school services, day or residential programs, individual and/or group support/therapy). When grouped by cognitive ability, 98% of LCA autistic adults and 44% of MCA autistic adults were reported by caregivers to have received at least one service in adulthood^3^. Consequently, the lower MCA endorsement of services as helpful may indicate reduced utilization or availability of these resources for this group, particularly in adulthood (Schott et al., 2020); however, further research is warranted. Findings from this study suggest that, from caregivers’ perspectives, these types of services remain helpful well into adulthood for LCA individuals and should continue to be prioritized and improved.

Further, verbally and cognitively more able autistic adults face numerous challenges in accessing higher education (Fernandes et al., 2021; Shattuck et al., 2012). The findings of this study emphasize the value of attending college or other forms of post-secondary education and provide some support for efforts to mitigate barriers to their educational attainment (Flegenheimer & Scherf, 2021; Scheef et al., 2019). Taken together, these differing patterns of helpful factors for MCA and LCA individuals, especially in adulthood, highlight the importance of considering both cognitive ability and phase of development in supporting these families.

The people in participants’ lives (i.e., family, professionals, friends/peers, and others) were endorsed as helpful significantly more frequently than specific services, aspects of education, and generative activities, which was true for both cognitive ability groups. This aligns with existing research highlighting the importance of relationships for autistic individuals, which evolve from childhood to adulthood, regardless of cognitive abilities (Chan et al., 2022; Losh et al., 2022; Robledo & Donnellan, 2016; Simplican et al., 2015; Smith et al., 2014). Although it was beyond the scope of the current study to examine specific types of people supports, the findings provide evidence that strong, positive relationships throughout the lifespan are a common value and priority across the spectrum of autistic individuals and their caregivers.

Similar patterns regarding the importance of generative activities (i.e., vocational/leisure activities and related internal/external factors) also emerged for both cognitive ability groups, with a clear increase in the endorsement of generative activities into adolescence and adulthood; an average 61% of the sample endorsed at least one generative activity as especially helpful in adulthood. This increase may be partly explained by the expanded opportunities such as employment and volunteering that become available or applicable within this timeframe. On the other hand, this increase could also be indicative of a greater need for engagement in community settings later in life, once autistic individuals no longer have structured activities provided by formal school systems (Gobbo & Shmulsky, 2016). These findings are consistent with previous work that has found community participation (e.g., community social groups, leisure and interest-based activities, work and volunteering) to be important from the perspective of autistic adults (Parenteau et al., 2023; Song et al., 2021). Relatively little attention in the autism research literature has been devoted to experiences of extracurricular, recreational, and/or community activities (Iwasa et al., 2022). However, given the importance of these types of activities highlighted by participants in this study, and the potential to mitigate symptoms of depression and serve as a context for meaningful social contact (Bishop-Fitzpatrick et al., 2017; Pappagianopoulos et al., 2023), expanding our understanding and clinical utility of generative activities, particularly in adulthood, is likely a fruitful direction for research.

### Negative Factors – Comparisons Between Cognitive Ability Groups

Where positive factors highlight diverse areas of strength, autistic adults’ and caregivers’ perceptions of negative factors across the lifespan reveal a “flip side” to these experiences. Overall, relatively similar patterns of negative factors were endorsed across cognitive ability groups and reporters for external social challenges (e.g., lack of social support/isolation, bullying/victimization), individual challenges (e.g., school/academic difficulties, social/adaptive skill barriers), medical/behavioral health challenges, adverse events, and factors that would have been helpful but were absent in the participants’ lives.

The congruence between some of these negative factors and positive factors described previously is striking. In particular, interpersonal relationships, school related factors, and engagement in service settings may be perceived as positive and beneficial to autistic individuals’ psychosocial development yet, under certain circumstances, can also pose significant challenges. For example, as demonstrated by participants’ reporting of positive people supports across their lives, interpersonal relationships can be a major developmental asset. At the same time, 23% of autistic adult self-reports in this study highlighted experiences of social isolation and negative peer social interactions. Prior work demonstrates that autistic youth and adults experience much greater rates of loneliness, social isolation, and peer victimization than their non-autistic peers (Libster et al., 2022; Lounds Taylor et al., 2024; Umagami et al., 2022). Similarly, in the current study, reporting of education-based factors as helpful by autistic adults and caregivers is juxtaposed with the 21% on average of the sample who reported school/academic difficulties as a significant hindrance. Indeed, existing research shows that autistic youth and caregivers frequently report negative educational experiences, including problems with sensory sensitivities, social expectations, and learning difficulties within the school setting (Gray et al., 2023; Kim et al., 2017).

Alongside these commonalities in identified support needs, distinctions observed in the endorsement of negative factors by cognitive ability also highlight unique considerations for these groups. That is, significantly more LCA caregivers reported challenges related to inadequately prepared professionals or caregivers and negative program experiences, whereas MCA caregivers more frequently identified family conflict and experiences of bullying or victimization as disruptive. As such, there is a need to enhance structured supports and service provisions for LCA autistic adults (e.g., access to autism-trained professionals who can deliver appropriate services and day programming), while for MCA autistic adults, increased interpersonal, family-based, and vocational supports and interventions emerge as especially important. Overall, this emphasizes the need for tailored care systems, which are responsive to autistic individuals’ cognitive and language abilities while also building on promotive factors and addressing related areas that require support and improvement.

### Limitations and Future Directions

Despite the strengths of this study, including a mixed-methods approach and assessment of direct reports of autistic adults and caregivers, there are several limitations to consider. Use of retrospective reporting across developmental stages covering nearly 30 years may limit accuracy of the data, particularly for autistic adults who were reporting on their own early years. Sample sizes also became relatively small when analyses were conducted by reporters (i.e., LCA caregivers, MCA caregivers, and MCA autistic adults) and in some cases by specific codes and subcategories. In addition, this is a unique sample of individuals who were diagnosed in early childhood in the 1990s and were followed longitudinally, with a majority being White and male. All of these factors impact the generalizability of the findings to the broader autism community.

Future work should build upon these findings to determine whether there are certain subtypes of services, people, education, and generative activities that emerge as more or less helpful to autistic individuals across the spectrum and across the lifespan. Collection of “current” promotive and disruptive factors data longitudinally may give different perspectives than retrospective reports. Furthermore, future research may wish to examine potential distinctions between participants’ ratings of factors based on their accessibility versus their utility, which was beyond the scope of this study.

## Conclusion

A recent Lancet review on the future of care and clinical research in autism (Lord et al., 2022) emphasizes the need for researchers and clinicians to adopt more individualized care approaches that account for the varying levels of support needed and accessed across different profiles of autistic individuals, their families, and their community settings, including those with co-occurring ID and profound support needs. The current study builds on this by identifying promotive and disruptive factors through direct reports from autistic individuals and their caregivers, underscoring the importance of considering differences across development and cognitive abilities. Over the reflected life stages in this study, services such as intervention or community programming became more beneficial for LCA autistic adults and education became increasingly helpful for MCA autistic adults. Findings indicate the importance of leveraging the people in autistic individuals’ lives–including family, friends, and professionals–to foster strong, supportive interpersonal relationships, regardless of ability or age period. Results also support a call for greater opportunities for safe exploration of interests and engagement with the community (i.e., generative activities), especially as autistic people of all cognitive abilities approach and navigate adulthood.

For clinicians and service providers, this may include intentional efforts to tailor interventions and support systems to meet the evolving needs of autistic individuals across developmental stages. For example, given the reported importance of generative activities across development, clinicians might facilitate goal setting in real-world contexts while helping to mitigate associated barriers. This could include supporting autistic youth in joining community-based organizations, peer support groups, or structured recreational activities, as well as exploring interest-based events, vocations, or activities.

For caregivers and educators, this may entail advocating for long-term service provisions that extend beyond early childhood and into adulthood, recognizing the critical role that both formal and informal support networks play in psychosocial development (Colver et al., 2019; Huang et al., 2022). Following autism evaluations, diagnosticians may refine recommendations for families by specifying individual service providers and making person-centered referrals whenever possible, rather than solely recommending types of therapies. This strategy emphasizes the importance of interpersonal compatibility in developing meaningful, long-term supports (Robledo & Donnellan, 2016). Additionally, caregivers, educators, service providers, and autistic individuals themselves may prioritize fostering independence through skill-building, self-advocacy, social engagement opportunities, and relationship-building across a range of community members. Furthermore, caregivers of LCA autistic individuals may also benefit from resources that improve access to specialized autism services and comprehensive training for care teams.

We hope these findings serve as an avenue for more individualized care and therapeutic approaches, ensuring that they are aligned with and relevant to the lived experiences of autistic individuals and their families.

## Electronic Supplementary Material

Below is the link to the electronic supplementary material.

Supplementary Material 1

## Footnotes

1 For parsimony, we reference this sample as autistic adults hereafter, rather than adults with autism and NDDs.

2 Two participants with VIQs 68 and 69 were characterized within the MCA group based on similar adaptive functioning as measured on the Vineland Adaptive Behavior Scales–Second Edition (VABS-II; Sparrow et al., 2005; MCA VABS-II Composite Score *M* = 79, *SD* = 17.32; LCA VABS-II Composite Score *M* = 32, *SD* = 14.29), as well as the nearly 20 point VIQ gap between these two scores and the next highest score within the LCA group.

3 Regarding service use as a whole, all participants in this study were recruited from provider referrals in childhood and most were receiving services starting at least at age 3 (see Anderson et al., 2014). As reported by caregivers, engagement in services was common during school age; however, the validity of the existing treatment data is limited by the lack of verification regarding whether the reported services were actively utilized while participants were in school.
