# Supplementary material for: What Factors Have Been the Most Helpful and Harmful and When? Identifying Key Impacts on Psychosocial Development According to Autistic Adults and Caregivers
Source: J Autism Dev Disord. 2025 Mar 27;56(9):3456–72. doi: 10.1007/s10803-025-06800-4 (PMC12327355; doi:10.1007/s10803-025-06800-4)
Supplement: Supplementary file 1 — Supplementary Material 1 [file 10803_2025_6800_MOESM1_ESM.docx]

**What Factors Have Been the Most Helpful and Harmful and When? Identifying Key Impacts on Psychosocial Development According to Autistic Adults and Caregivers**

Juliette E. Lerner^1^, Hillary Schiltz^2^, Noa Schisterman^1^, Sonja Ziegler^3^, & Catherine Lord^1^

*Journal of Autism and Developmental Disorders*

^1^University of California, Los Angeles, Semel Institute for Neuroscience and Human Behavior, 760 Westwood Plaza, Suite 68-217, Los Angeles, CA, 90024, United States of America

^2^University of Washington, Department of Pediatrics in the Institute on Human Development and Disability, 1701 NE Columbia Road, Seattle, WA, 98195, United States of America

^3^Research Unit of Child and Adolescent Mental Health, Department of Clinical Research, University of Southern Denmark, J.B Winsløws Vej 16, indgang 230, 5000, Odense, Denmark

*Corresponding author*: Correspondence concerning this article should be addressed to Juliette Lerner, Semel Institute for Neuroscience and Human Behavior, University of California, Los Angeles, 760 Westwood Plaza, Los Angeles, CA 90024, USA, [JELerner@mednet.ucla.edu](mailto:JELerner@mednet.ucla.edu)

**Online Resource 1**

*Percentage of Endorsed Positive Factors Reflecting Across Developmental Stages by Reporter*

| **Categories, Subcategories, & Codes** | **Positive Factors** | | | | | | | |
| --- | --- | --- | --- | --- | --- | --- | --- | --- |
| **Category: Services** | | | | | | | | |
|  | **Early Childhood** | | | | **School Age** | | | |
|  | All  *n* = 100 | LCA Caregiver  *n* = 42 | MCA Caregiver  *n* = 32 | MCA Adult  *n* = 26 | All  *n* = 112 | LCA Caregiver  *n* = 42 | MCA Caregiver  *n* = 37 | MCA Adult  *n* = 33 |
|  | % | % | % | % | % | % | % | % |
| ***Subcategory: Intervention*** | 38.00 | 40.48 | 46.88 | 23.08 | 18.75 | 23.81 | 18.92 | 12.12 |
| *Applied Behavior Analysis (ABA)* | 8.00 | 16.67 | 3.13 | 3.85 | 5.36 | 11.90 | 2.70 | 0.00 |
| *Speech Therapy* | 16.00 | 11.90 | 31.25 | **^†^3.85** | 6.25 | 4.76 | 10.81 | 3.03 |
| *Occupational/Physical Therapy* | 14.00 | 11.90 | 25.00 | **^†^3.85** | 5.36 | 4.76 | 10.81 | 0.00 |
| *TEACCH Program* | 7.00 | 9.52 | 6.25 | 3.85 | 2.68 | 4.76 | 0.00 | 3.03 |
| *Other Intervention/Desensitization/Intv. Unspecified* | 13.00 | 9.52 | 21.88 | 7.69 | 8.04 | 7.14 | 10.81 | 6.06 |
| ***Subcategory: Assessment*** | 7.00 | 2.38 | 12.59 | 7.69 | 0.00 | 0.00 | 0.00 | 0.00 |
| *Early Autism Diagnosis* | 5.00 | 2.38 | 9.38 | 3.85 | 0.00 | 0.00 | 0.00 | 0.00 |
| *Autism Evaluation/Assessment Unspecified* | 2.00 | 0.00 | 3.13 | 3.85 | 0.00 | 0.00 | 0.00 | 0.00 |
| ***Subcategory: Other Program/Community Resources*** | 13.00 | 16.67 | 12.59 | 7.69 | 13.39 | **26.19** | 8.11 | 3.03 |
| *Community-Based/Day Program/Program Unspec.* | 8.00 | 11.90 | 6.25 | 3.85 | 7.14 | **19.05** | 0.00 | 0.00 |
| *Accommodations/Academic Support* | 1.00 | 0.00 | 0.00 | 3.85 | 4.46 | 4.78 | 5.41 | 3.03 |
| *Medical Services* | 4.00 | 4.76 | 5.41 | 0.00 | 1.79 | 2.38 | 2.70 | 0.00 |
|  | **Adolescence** | | | | **Adulthood** | | | |
|  | All  *n* = 104 | LCA Caregiver  *n* = 38 | MCA Caregiver  *n* = 34 | MCA Adult  *n* = 32 | All  *n* = 112 | LCA Caregiver  *n* = 42 | MCA Caregiver  *n* = 35 | MCA Adult  *n* = 35 |
| ***Subcategory: Programming*** | 13.46 | **28.95** | 8.82 | 0.00 | 23.21 | **52.38** | 5.71 | 5.71 |
| *Intervention/Desensitization* | 7.69 | 13.16 | 8.82 | 0.00 | 2.68 | 4.76 | 0.00 | 2.86 |
| *Community-Based/Day Program/Program Unspec.* | 5.77 | **15.79** | 0.00 | 0.00 | 17.86 | **42.86** | 2.86 | 2.86 |
| *Group Home/Residential Program* | 0.00 | 0.00 | 0.00 | 0.00 | 6.25 | 14.29 | 2.86 | 0.00 |
| ***Subcategory: Educational Resources*** | 10.58 | 7.89 | 17.65 | 6.25 | 16.07 | 19.05 | 17.14 | 11.43 |
| *Skills Courses (e.g., life, social)* | 3.85 | 5.26 | 2.94 | 3.13 | 2.68 | 7.14 | 0.00 | 0.00 |
| *Transition/Vocational Program* | 3.85 | 0.00 | 8.82 | 3.13 | 11.61 | 11.90 | 14.29 | 8.57 |
| *Accommodations/Academic Support* | 2.88 | 2.63 | 5.88 | 0.00 | 2.68 | 0.00 | 5.71 | 2.86 |
| ***Subcategory: Medical Resources*** | 3.85 | 5.26 | 0.00 | 6.25 | 3.57 | 7.14 | 2.86 | 0.00 |
| *Medical Services* | 0.96 | 0.00 | 0.00 | 3.13 | 2.68 | 4.76 | 2.86 | 0.00 |
| *Alternative Medicine* | 2.88 | 5.26 | 0.00 | 3.13 | 0.89 | 2.38 | 0.00 | 0.00 |
| **Category: People** | | | | | | | | |
|  | **Early Childhood** | | | | **School Age** | | | |
|  | All  *n* = 100 | LCA Caregiver  *n* = 42 | MCA Caregiver  *n* = 32 | MCA Adult  *n* = 26 | All  *n* = 112 | LCA Caregiver  *n* = 42 | MCA Caregiver  *n* = 37 | MCA Adult  *n* = 33 |
|  | % | % | % | % | % | % | % | % |
| ***Subcategory: Family*** | 48.00 | 28.57 | **59.38** | 65.38 | 31.25 | 21.43 | 37.84 | 36.36 |
| *Parents* | 22.00 | 9.52 | 21.88 | 42.30 | 15.18 | 7.14 | 18.92 | 21.21 |
| *Grandparents/Great Grandparents* | 9.00 | 7.14 | 12.50 | 7.69 | 3.57 | 7.14 | 0.00 | 3.03 |
| *Siblings* | 17.00 | 7.14 | **31.25** | 15.38 | 8.93 | 4.76 | 16.22 | 6.06 |
| *Family in General/Family Environment* | 14.00 | 9.52 | 15.63 | 19.23 | 8.04 | 4.76 | 10.81 | 9.09 |
| *Other Family Members* | 2.00 | 0.00 | 6.25 | 0.00 | 1.79 | 0.00 | 5.41 | 0.00 |
| ***Subcategory: Formal Supports*** | 27.00 | 34.15 | 31.25 | 7.69 | 39.29 | 52.38 | 32.43 | 27.27 |
| *Teachers/Professors/Principal* | 6.00 | 9.52 | 6.25 | 0.00 | 23.21 | 33.33 | 18.92 | 15.15 |
| *Professionals/Support Staff/Therapist* | 25.00 | 33.33 | 28.13 | 7.69 | 18.75 | 23.81 | 18.92 | 12.12 |
| ***Subcategory: Informal Supports*** | 14.00 | 14.29 | 15.63 | 11.54 | 17.86 | 14.29 | 21.62 | 18.18 |
| *Friends/Peers/Social Environment* | 12.00 | 11.90 | 12.50 | 11.54 | 13.39 | 7.14 | 18.92 | 15.15 |
| *Specifically Named Unknown Person/People* | 4.00 | 4.76 | 6.25 | 0.00 | 4.46 | 7.14 | 2.70 | 3.03 |
|  | **Adolescence** | | | | \  **Adulthood** | | | |
|  | All  *n* = 104 | LCA Caregiver  *n* = 38 | MCA Caregiver  *n* = 34 | MCA Adult  *n* = 32 | All  *n* = 112 | LCA Caregiver  *n* = 42 | MCA Caregiver  *n* = 35 | MCA Adult  *n* = 35 |
| ***Subcategory: Family*** | 28.85 | 26.32 | 29.41 | 31.25 | 25.89 | 30.95 | 20.00 | 25.71 |
| *Parents* | 14.42 | 7.89 | 14.71 | 21.86 | 10.71 | 7.14 | 11.43 | 14.29 |
| *Grandparents/Great Grandparents* | 3.85 | 10.53 | 0.00 | 0.00 | 3.57 | 4.76 | 0.00 | 5.71 |
| *Siblings* | 9.62 | 2.63 | **17.65** | 9.38 | 11.61 | 11.90 | 14.29 | 8.57 |
| *Family in General/Family Environment* | 9.62 | 10.53 | 8.82 | 9.38 | 7.14 | 14.29 | 2.86 | 2.86 |
| *Other Family Members* | 0.00 | 0.00 | 0.00 | 0.00 | 3.57 | 7.14 | 0.00 | 2.86 |
| ***Subcategory: Formal Supports*** | 36.54 | **^†^50.00** | 26.47 | 31.26 | 21.43 | 33.33 | 22.86 | 5.71 |
| *Teachers/Professors/Principal* | 23.08 | 28.95 | 14.71 | 25.00 | 3.57 | 2.38 | 8.57 | 0.00 |
| *Professionals/Support Staff/Therapist* | 17.31 | 28.95 | 17.65 | 3.13 | 13.39 | 26.19 | 8.57 | 2.86 |
| *Coaches/Mentors* | 3.85 | 0.00 | 8.82 | 3.13 | 6.25 | 7.14 | 8.57 | 2.86 |
| *Other Caregiver/Respite Provider* | 3.85 | 10.53 | 0.00 | 0.00 | 2.68 | 4.76 | 2.86 | 0.00 |
| ***Subcategory: Informal Supports*** | 33.65 | 28.95 | 41.18 | 31.25 | 30.35 | 14.29 | **42.86** | 37.14 |
| *Friends/Peers/Social Environment* | 29.81 | 26.32 | 38.24 | 25.00 | 13.39 | 9.52 | 17.14 | 14.29 |
| *Romantic Partner/Spouse* | 0.00 | 0.00 | 0.00 | 0.00 | 7.14 | 0.00 | **11.43** | 11.43 |
| *Coworkers/Managers* | 0.00 | 0.00 | 0.00 | 0.00 | 1.79 | 0.00 | 5.71 | 0.00 |
| *Animals (service and pets)* | 0.00 | 0.00 | 0.00 | 0.00 | 2.68 | 0.00 | 0.00 | 8.57 |
| *Specifically Named Unknown Person/People* | 4.81 | 5.26 | 2.94 | 6.25 | 9.82 | 4.76 | 14.29 | 11.43 |
| **Category: Education** | | | | | | | | |
|  | **Early Childhood** | | | | **School Age** | | | |
|  | All  *n* = 100 | LCA Caregiver  *n* = 42 | MCA Caregiver  *n* = 32 | MCA Adult  *n* = 26 | All  *n* = 112 | LCA Caregiver  *n* = 42 | MCA Caregiver  *n* = 37 | MCA Adult  *n* = 33 |
|  | % | % | % | % | % | % | % | % |
| ***Subcategory: School Characteristics*** | 11.00 | 14.29 | 12.50 | 3.85 | 16.07 | 19.05 | 21.62 | 6.06 |
| *Special Education/Autism Specific Program* | 9.00 | 9.52 | 12.50 | 3.85 | 9.82 | 14.29 | 8.11 | 6.06 |
| *General Education/Integrated in General Student Pop.* | 3.00 | 7.14 | 0.00 | 0.00 | 4.46 | 4.76 | 8.11 | 0.00 |
| *Smaller Classroom/Environment* | 0.00 | 0.00 | 0.00 | 0.00 | 2.68 | 2.38 | 5.41 | 0.00 |
| ***Subcategory: School Type*** | 2.00 | 0.00 | 3.13 | 3.85 | 6.25 | 2.38 | 10.81 | 6.06 |
| *Homeschool* | 1.00 | 0.00 | 0.00 | 3.85 | 2.68 | 0.00 | 5.41 | 3.03 |
| *Private School* | 1.00 | 0.00 | 3.13 | 0.00 | 3.57 | 2.38 | 5.41 | 3.03 |
| ***Subcategory: Schooling/Learning in General*** | 11.00 | 11.90 | 12.50 | 7.69 | 13.39 | 14.29 | 13.51 | 12.12 |
| *Schooling/Education in General* | 8.00 | 7.14 | 9.38 | 7.69 | 11.61 | 14.29 | 10.81 | 9.09 |
| *Academic Skill Learning/Development* | 3.00 | 4.76 | 3.13 | 0.00 | 1.79 | 0.00 | 2.70 | 3.03 |
|  | **Adolescence** | | | | **Adulthood** | | | |
|  | All  *n* = 104 | LCA Caregiver  *n* = 38 | MCA Caregiver  *n* = 34 | MCA Adult  *n* = 32 | All  *n* = 112 | LCA Caregiver  *n* = 42 | MCA Caregiver  *n* = 35 | MCA Adult  *n* = 35 |
| ***Subcategory: School Characteristics*** | 11.54 | 15.79 | 8.82 | 9.38 | 0.00 | 0.00 | 0.00 | 0.00 |
| *Special Ed/Autism Specific Program* | 6.73 | 5.26 | 5.88 | 9.38 | 0.00 | 0.00 | 0.00 | 0.00 |
| *General Education/Integrated* | 4.81 | 7.89 | 5.88 | 0.00 | 0.00 | 0.00 | 0.00 | 0.00 |
| *Smaller Classroom/Environment* | 1.92 | 2.63 | 2.94 | 0.00 | 0.00 | 0.00 | 0.00 | 0.00 |
| ***Subcategory: School Type*** | 3.84 | 2.63 | 5.88 | 3.13 | 0.00 | 0.00 | 0.00 | 0.00 |
| *Homeschool* | 1.92 | 0.00 | 2.94 | 3.13 | 0.00 | 0.00 | 0.00 | 0.00 |
| *Private School* | 1.92 | 2.63 | 2.94 | 0.00 | 0.00 | 0.00 | 0.00 | 0.00 |
| ***Subcategory: Schooling/Learning in General*** | 20.19 | 18.42 | 17.65 | 25.00 | 33.93 | 11.90 | **54.29** | 40.00 |
| *Schooling/Education/Post-Secondary Ed/Degree* | 16.35 | 15.79 | 17.65 | 15.63 | 28.57 | 7.14 | **45.71** | 37.14 |
| *Academic Skill Learning/Development* | 4.81 | 2.63 | 2.94 | 9.36 | 6.25 | 4.76 | 11.43 | 2.86 |
| **Category: Generative Activities** | | | | | | | | |
|  | **Early Childhood** | | | | **School Age** | | | |
|  | All  *n* = 100 | LCA Caregiver  *n* = 42 | MCA Caregiver  *n* = 32 | MCA Adult  *n* = 26 | All  *n* = 112 | LCA Caregiver  *n* = 42 | MCA Caregiver  *n* = 37 | MCA Adult  *n* = 33 |
|  | % | % | % | % | % | % | % | % |
| ***Subcategory: Vocational/Leisure Activities*** | 13.00 | 11.90 | 9.38 | 19.23 | 19.64 | 11.90 | 16.23 | 33.33 |
| *Hobbies/Extra Curriculars/Community Activities* | 13.00 | 11.90 | 9.38 | 19.23 | 19.64 | 11.90 | 16.23 | 33.33 |
| ***Subcategory: Individual/Internal Factors*** | 4.00 | 4.76 | 3.13 | 3.85 | 4.46 | 4.76 | 2.70 | 6.06 |
| *Developing Autonomy/Independence/Identity* | 0.00 | 0.00 | 0.00 | 0.00 | 2.68 | 2.38 | 2.70 | 3.03 |
| *Social Communication Skills/Sign Language* | 4.00 | 4.76 | 3.13 | 3.85 | 2.68 | 2.38 | 2.70 | 3.03 |
| ***Subcategory: Environmental/External Factors*** | 5.00 | 9.52 | 3.13 | 0.00 | 4.46 | 9.52 | 2.70 | 0.00 |
| *Structure/Routine* | 1.00 | 2.38 | 0.00 | 0.00 | 3.57 | 4.76 | 0.00 | 0.00 |
| *Transportation* | 1.00 | 2.38 | 0.00 | 0.00 | 0.00 | 0.00 | 0.00 | 0.00 |
| *Religious Institution/Church* | 3.00 | 4.76 | 3.13 | 0.00 | 2.68 | 4.76 | 2.70 | 0.00 |
|  | **Adolescence** | | | | **Adulthood** | | | |
|  | All  *n* = 104 | LCA Caregiver  *n* = 38 | MCA Caregiver  *n* = 34 | MCA Adult  *n* = 32 | All  *n* = 112 | LCA Caregiver  *n* = 42 | MCA Caregiver  *n* = 35 | MCA Adult  *n* = 35 |
| ***Subcategory: Vocational/Leisure Activities*** | 50.00 | 39.47 | 44.12 | 46.86 | 43.75 | 28.57 | **57.14** | 48.57 |
| *Hobbies/Extra Curriculars/Travel/Community Activities* | 39.42 | 31.58 | 41.12 | 46.88 | 22.32 | 23.81 | 22.86 | 20.00 |
| *Employment/Career/Volunteer Work* | 5.77 | 7.89 | 8.82 | 0.00 | 25.00 | 7.14 | **37.14** | 34.29 |
| ***Subcategory: Individual/Internal Factors*** | 8.65 | 7.89 | 2.94 | 15.63 | 16.07 | 7.14 | 22.86 | 20.00 |
| *Developing Autonomy/Independence/Identity* | 5.77 | 5.26 | 2.94 | 9.36 | 15.18 | 7.14 | 20.00 | 20.00 |
| *Individual Trait/Outlook/Attitude/Intrinsic Motivation* | 2.88 | 2.61 | 0.00 | 6.25 | 0.89 | 0.00 | 2.86 | 0.00 |
| ***Subcategory: Environmental/External Factors*** | 8.65 | 13.16 | 5.26 | 6.25 | 25.89 | 30.95 | 22.86 | 22.86 |
| *Structure/Routine* | 0.96 | 0.00 | 0.00 | 3.13 | 8.04 | **19.00** | 2.86 | 0.00 |
| *Transportation* | 0.00 | 0.00 | 0.00 | 0.00 | 1.79 | 0.00 | 5.71 | 0.00 |
| *Religious Institution/Church* | 5.77 | 10.53 | 2.94 | 3.13 | 5.36 | 7.14 | 8.57 | 0.00 |
| *Moving Out* | 0.00 | 0.00 | 0.00 | 0.00 | 3.57 | 2.38 | 0.00 | 8.57 |
| *Finances* | 0.00 | 0.00 | 0.00 | 0.00 | 4.46 | 2.38 | 2.86 | 8.57 |
| *Access and Info About Services* | 0.96 | 2.63 | 0.00 | 0.00 | 3.57 | 7.14 | 2.86 | 0.00 |
| *Technology* | 0.96 | 0.00 | 2.63 | 0.00 | 4.46 | 4.76 | 2.86 | 5.71 |

*Note.* **Bold** indicates significantly greater endorsement of subcategory or code between LCA and MCA caregiver reports at *p* < .05, **^†^bold** indicates at *p* = .054 for the difference between LCA and MCA caregiver reports and at *p* = .063 for the differences between MCA caregiver and MCA autistic adult reports; missing or un-codable data varied from 9%-19%; sample sizes and percentages reported above are excluding missing and un-codable data. MCA = more cognitively able; LCA = less cognitively able. TEACCH = Treatment and Education of Autistic and related Communication-handicapped CHildren.

**Online Resource 2**

*Multilevel Logistic Regression Model Fixed Effects on Positive Factors Endorsement*

|  | *Model Terms* | *F* | *df1* | *df2* | *p* |
| --- | --- | --- | --- | --- | --- |
| **Model 1: LCA vs. MCA Caregiver Report** | Corrected Model | 5.30 | 31 | 1176 | < .001 |
|  | Developmental Stage | 1.30 | 3 | 1176 | .274 |
|  | Positive Factors Category | 32.64 | 3 | 1176 | < .001 |
|  | LCA vs. MCA | .11 | 1 | 1176 | .740 |
|  | Developmental Stage x Positive Factors Category | 5.33 | 9 | 1176 | < .001 |
|  | LCA vs. MCA Caregiver x Developmental Stage x Positive Factors Category | 2.27 | 15 | 1176 | .004 |
| **Model 2: MCA Autistic Adult vs. MCA Caregiver Report** | Corrected Model | 5.61 | 31 | 1015 | < .001 |
|  | Developmental Stage | 2.23 | 3 | 1015 | .083 |
|  | Positive Factors Category | 36.59 | 3 | 1015 | < .001 |
|  | MCA Autistic Adult vs. Caregiver | 2.29 | 1 | 1015 | .130 |
|  | Developmental Stage x Category | 6.25 | 9 | 1015 | < .001 |
|  | MCA Autistic Adult vs. Caregiver x Positive Factors Category x Developmental Stage | 1.22 | 15 | 1015 | .249 |

*Note*. Endorsement refers to a binary code indicating whether at least one positive factor code (i.e., within *services*, *people*, *education*, or *generative activities*) was endorsed versus not endorsed. Developmental stage = early childhood, school age, adolescence, or adulthood; positive factors category = *services*, *people*, *education*, or *generative activities*. All variables are categorical and use dummy coding. LCA = less cognitively able; MCA = more cognitively able.

**Online Resource 3**

*Positive Factors Multilevel Model Fixed Effects Coefficients*

| **Predictor** | **Estimate (Exp(β))** | ***SE*** | **95% CI** | ***p*** |
| --- | --- | --- | --- | --- |
|  |  |  |  |  |
| **Model 1: LCA vs. MCA Caregiver Report** |  |  |  |  |
| Intercept | 1.91 | 0.36 | [0.95, 3.87] | .071 |
| Developmental Stage |  |  |  |  |
| - Early Childhood = 1 | 0.10 | 0.60 | [0.03, 0.32] | < .001 |
| - School Age = 2 | 0.14 | 0.54 | [0.05, 0.41] | < .001 |
| - Adolescence = 3 | 0.46 | 0.50 | [0.18, 1.22] | .121 |
| - Adulthood (reference) = 4 | — | — | — | — |
| Positive Factors Category |  |  |  |  |
| - Services = 1 | 0.18 | 0.53 | [0.06, 0.51] | .001 |
| - People = 2 | 0.88 | 0.50 | [0.33, 2.35] | .803 |
| - Education = 3 | 0.62 | 0.49 | [0.24, 1.63] | .330 |
| - Generative Activities (reference) = 4 | — | — | — | — |
| LCA vs. MCA |  |  |  |  |
| - LCA | 0.58 | 0.48 | [0.23, 1.46] | .246 |
| - MCA (reference) | — | — | — | — |
| Interaction Terms |  |  |  |  |
| Early Childhood x Services | 43.99 | 0.80 | [9.11, 212.39] | < .001 |
| Early Childhood x People | 18.44 | 0.81 | [3.77, 90.11] | < .001 |
| Early Childhood x Education | 3.42 | 0.80 | [0.72, 16.31] | .123 |
| School Age x Services | 7.46 | 0.76 | [1.69, 32.97] | .008 |
| School Age x People | 9.74 | 0.73 | [2.31, 41.13] | .002 |
| School Age x Education | 4.00 | 0.72 | [0.98, 16.33] | .054 |
| Adolescence x Services | 1.92 | 0.75 | [0.44, 8.32] | .383 |
| Adolescence x People | 3.06 | 0.71 | [0.75, 12.43] | .117 |
| Adolescence x Education | 0.87 | 0.70 | [0.22, 3.45] | .841 |
| LCA x Early Childhood x Services | 1.43 | 0.67 | [0.39, 5.35] | .590 |
| LCA x Early Childhood x People | 1.04 | 0.70 | [0.26, 4.13] | .957 |
| LCA x Early Childhood x Education | 1.38 | 0.71 | [0.34, 5.61] | .650 |
| LCA x Early Childhood x Generative Activities | 2.93 | 0.77 | [0.65, 13.24] | .163 |
| LCA x School Age x Services | 3.50 | 0.68 | [0.93, 13.22] | .064 |
| LCA x School Age x People | 2.06 | 0.69 | [0.53, 7.99] | .294 |
| LCA x School Age x Education | 1.27 | 0.67 | [0.34, 4.67] | .722 |
| LCA x School Age x Generative Activities | 2.22 | 0.71 | [0.55, 8.98] | .262 |
| LCA x Adolescence x Services | 3.66 | 0.71 | [0.92, 14.61] | .066 |
| LCA x Adolescence x People | 3.19 | 0.74 | [0.75, 13.50] | .115 |
| LCA x Adolescence x Education | 1.88 | 0.69 | [0.49, 7.25] | .361 |
| LCA x Adolescence x Generative Activities | 1.94 | 0.67 | [0.52, 7.21] | .321 |
| LCA x Adulthood x Services | 9.10 | 0.69 | [2.35, 35.25] | .001 |
| LCA x Adulthood x People | 1.52 | 0.67 | [0.41, 5.60] | .533 |
| LCA x Adulthood x Education | 0.20 | 0.75 | [0.05, 0.86] | .031 |

| **Model 2: MCA Autistic Adult vs. MCA Caregiver Report** |  |  |  |  |
| --- | --- | --- | --- | --- |
| Intercept | 1.92 | 0.36 | [0.95, 3.86] | .069 |
| Developmental Stage |  |  |  |  |
| - Early Childhood = 1 | 0.12 | 0.61 | [0.04, 0.41] | .001 |
| - School Age = 2 | 0.34 | 0.50 | [0.13, 0.91] | .032 |
| - Adolescence = 3 | 1.15 | 0.52 | [0.41, 3.20] | .791 |
| - Adulthood (reference) = 4 | — | — | — | — |
| Positive Factors Category |  |  |  |  |
| - Services = 1 | 0.09 | 0.60 | [0.03, 0.28] | < .001 |
| - People = 2 | 0.78 | 0.50 | [0.30, 2.07] | .621 |
| - Education = 3 | 0.35 | 0.50 | [0.13, 0.92] | .033 |
| - Generative Activities (reference) = 4 | — | — | — | — |
| MCA Autistic Adult vs. MCA Caregiver |  |  |  |  |
| - MCA Caregiver | 1.00 | 0.51 | [0.37, 2.69] | .999 |
| - MCA Autistic Adult (reference) | — | — | — | — |
| Interaction Terms |  |  |  |  |
| Services x Early Childhood | 21.49 | 0.89 | [3.76, 122.75] | .001 |
| Services x School Age | 3.16 | 0.85 | [0.60, 16.77] | .176 |
| Services x Adolescence | 0.75 | 0.89 | [0.13, 4.28] | .743 |
| People x Early Childhood | 17.91 | 0.84 | [3.43, 93.62] | .001 |
| People x School Age | 4.52 | 0.72 | [1.10, 18.53] | .036 |
| People x Adolescence | 1.48 | 0.74 | [0.35, 6.33] | .593 |
| Education x Early Childhood | 2.20 | 0.89 | [0.38, 12.55] | .376 |
| Education x School Age | 1.16 | 0.79 | [0.25, 5.47] | .853 |
| Education x Adolescence | 0.68 | 0.73 | [0.16, 2.86] | .603 |
| MCA Autistic Adult vs. MCA Caregiver x Services x Early Childhood | 3.31 | 0.75 | [0.76, 14.44] | .112 |
| MCA Autistic Adult vs. MCA Caregiver x Services x School Age | 2.08 | 0.79 | [0.44, 9.84] | .355 |
| MCA Autistic Adult vs. MCA Caregiver x Services x Adolescence | 2.15 | 0.84 | [0.42, 11.16] | .361 |
| MCA Autistic Adult vs. MCA Caregiver x Services x Adulthood | 2.08 | 0.80 | [0.43, 9.94] | .360 |
| MCA Autistic Adult vs. MCA Caregiver x People x Early Childhood | 0.90 | 0.80 | [0.19, 4.33] | .900 |
| MCA Autistic Adult vs. MCA Caregiver x People x School Age | 1.03 | 0.73 | [0.25, 4.28] | .966 |
| MCA Autistic Adult vs. MCA Caregiver x People x Adolescence | 0.94 | 0.74 | [0.22, 4.02] | .932 |
| MCA Autistic Adult vs. MCA Caregiver x People x Adulthood | 1.13 | 0.70 | [0.28, 4.49] | .864 |
| MCA Autistic Adult vs. MCA Caregiver x Education x Early Childhood | 2.16 | 0.84 | [0.42, 11.23] | .358 |
| MCA Autistic Adult vs. MCA Caregiver x Education x School Age | 2.61 | 0.79 | [0.56, 12.24] | .222 |
| MCA Autistic Adult vs. MCA Caregiver x Education x Adolescence | 0.91 | 0.73 | [0.22, 3.79] | .900 |
| MCA Autistic Adult vs. MCA Caregiver x Education x Adulthood | 1.78 | 0.70 | [0.45, 7.02] | .409 |
| MCA Autistic Adult vs. MCA Caregiver x Generative Activities x Early Childhood | 0.78 | 0.86 | [0.14, 4.22] | .775 |
| MCA Autistic Adult vs. MCA Caregiver x Generative Activities x School Age | 0.43 | 0.74 | [0.10, 1.80] | .246 |
| MCA Autistic Adult vs. MCA Caregiver x Generative Activities x Adolescence | 0.40 | 0.72 | [0.10, 1.66] | .208 |

*Note.* Exp(β) refers to the exponentiated coefficient (odds ratio). CI = confidence interval; *SE* = standard error. The reference category for categorical variables is specified in parentheses. LCA = less cognitively able; MCA = more cognitively able.
